# The viable but nonculturable state induction and genomic analyses of *Lactobacillus casei* BM‐LC14617, a beer‐spoilage bacterium

**DOI:** 10.1002/mbo3.506

**Published:** 2017-07-06

**Authors:** Junyan Liu, Lin Li, Brian M. Peters, Bing Li, Lequn Chen, Yang Deng, Zhenbo Xu, Mark E. Shirtliff

**Affiliations:** ^1^ School of Food Science and Engineering South China University of Technology Guangzhou China; ^2^ Department of Clinical Pharmacy College of Pharmacy University of Tennessee Health Science Center Memphis Tennessee; ^3^ Guangdong Province Key Laboratory for Green Processing of Natural Products and Product Safety Guangzhou China; ^4^ Department of Microbial Pathogenesis School of Dentistry University of Maryland Baltimore Maryland

**Keywords:** beer‐spoilage, genomic sequencing, *Lactobacillus casei*, VBNC state

## Abstract

This study aimed to investigate the viable but nonculturable (VBNC) state and genomic features of a beer‐spoilage strain, *Lactobacillus casei* BM‐LC14617. Induction on the VBNC state of *L*. *casei* strain BM‐LC14617 was conducted by both low‐temperature storage and continuous passage in beer, and formation of VBNC state was detected after 196 ± 3.3 days and 32 ± 1.6 subcultures, respectively. Resuscitation of VBNC cells was successfully induced by addition of catalase, and culturable, VBNC, and resuscitated cells shared similar beer‐spoilage capability. Whole genome sequencing was performed, and out of a total of 3,964 predicted genes, several potential VBNC and beer‐spoilage‐associated genes were identified. *L*. *casei* is capable of entering into and resuscitating from the VBNC state and possesses beer‐spoilage capability. The genomic characterization yield insightful elucidation of VBNC state for *L*. *casei*. This study represents the first evidence on VBNC state induction of L. casei and beer‐spoilage capability of VBNC and resuscitated cells. Also, this is the first genomic characterization of L. casei as a beer‐spoilage bacterium. The current study may aid in further study on L. casei and other beer‐spoilage bacteria, and guide the prevention and control of beer spoilage.

## 1 INTRODUCTION

As a popular beverage, beer has been recognized as safe due to its high microbiological stability, as a large variety of microorganisms are incapable of survival in beer (Sakamoto & Konings, 2003; Suzuki, Iijima, Asano, Kuriyama, & Kitagawa, 2006). However, despite these unfavorable conditions for microbial growth, a few species of bacteria (primarily Lactobacilli and Pediococci) remain viable in this medium and are designated as beer‐spoilage microorganisms. A number of lactic acid bacteria (LAB) have been well documented as a major cause of acidity and turbidity in beer. As an important beer‐spoilage bacterium, *Lactobacillus casei* possesses the ability to produce lactic acid, acetic acid, and diacetyl as end or by‐products of carbohydrate fermentation, thus significantly influencing the flavor of beer. This issue is further complicated by the fact that contaminating *L*. *casei* strains are able to cause false‐negative detection, due to their capability of entering into the viable but noncluturable (VBNC) state under stress conditions (Deng et al., 2015; Suzuki et al., 2006). Bacteria in the VBNC state fail to grow on the routine bacteriological media on which they would normally grow and develop into colonies, but still live and have metabolic activity (Oliver, 2005).

This study aimed to investigate the VBNC state and genomic features through genomic sequencing of *L*. *casei* strain BM‐LC14617 as a beer‐spoilage bacterium.

## 2 MATERIALS AND METHODS

### 2.1 Bacterial strains and growth conditions

*L*. *casei* strain, designated BM‐LC14617, was isolated from finished beer and grown anaerobically at 26°C in MRS broth (Oxoid, UK).

### 2.2 Induction of entry into VBNC state

Approximately 10^7^ cells/ml exponentially growing cells of *L*. *casei* strain BM‐LC14617 were inoculated and anaerobically subcultured at 26°C in 10 ml aliquots of the degassed commercial beer. Afterward, the bacterial strain was induced to entry into VBNC state by both low‐temperature storage and continuous passage in beer. Continuous passage in beer was performed as described by Suzuki et al. (2006) with some modification. The interval of each subculture was 7 days. Concerning the low‐temperature storage, the exponentially growing cells of *L*. *casei* BM‐LC14617 were harvested by centrifugation at 5000 x g for 15 min at 4°C. The bacterial cells were then resuspended in degassed beer at a final density of 10^7^ cells/ml and maintained at 0°C without shaking to induce the VBNC state.

### 2.3 Culturability and viability assays

In this study, the term “VBNC state” was defined as the inability to grow on MRS agar media until 14 days of incubation. The number of culturable cells were accessed by a conventional plate culture procedure (Suzuki, Asano, Iijima, & Kitamoto, 2008). A portion of subcultures (100 μL) was inoculated on MRS agar and incubated anaerobically at 26°C. The inoculated agar plate was examined each day for the presence of colony formation, and the day on which colonies were first observed was recorded as the detection time. After 14 days of incubation, CFU were counted to determine culturability on agar plates. The number of total cells was determined by AODC method (Hobbie, Daley, & Jasper, 1977). Cell viability was determined using a Live/Dead BacLight bacterial viability kit (Molecular Probes, USA) with fluorescent microscope (Berney, Hammes, Bosshard, Weilenmann, & Egli, 2007). Cellular viability and culturability were evaluated once a week.

### 2.4 Resuscitation of the VBNC state

After entry into the VBNC state, 100 μL of *L*. *casei* cells were inoculated in 10 ml of MRS broth or sterile water (negative control), respectively. Adding chemicals (Tween‐20, tween‐80, vitamin C, vitamin B2, and catalase) (Sigma‐Aldrich, USA) were attempted to resuscitate *L*. *casei*. Simultaneously, heat‐denatured catalase (60°C for 15 min) served as a control. The culturability of the samples was determined by plating 100 μL of the samples on MRS agar.

### 2.5 Evaluation of beer‐spoilage ability

The beer‐spoilage ability was investigated using the traditional “growth in beer test” (Sakamoto & Konings, 2003). Approximately 10^5^ cells/ml of the strain in exponentially growing, VBNC, and resuscitated state were inoculated into commercial bottled lager beer under sterile conditions at room temperature, respectively. The inoculated beer were incubated at 26°C and examined daily for visible growth for up to 30 days. Subsequently, lactic acid and acetic acid concentration were analyzed by reversed‐phase high performance liquid chromatography. The diacetyl concentration was measured by Head Space Gas Chromatography. Lactic acid, acetic acid, and diacetyl were quantified by the external standard method.

### 2.6 Whole genome sequencing, assembly, and annotation

The genome of *L*. *casei* BM‐LC14617 was sequenced using the Illumina HiSeq 2,500 platform and paired‐end libraries. The K‐mer length was 17 and the qualities of the reads were examined by FastQC v.0.10.1 (http://www.bioinformatics.babraham.ac.uk/projects/fastqc/) before assembly. Filtered sequences were assembled de novo using Velvet v1.2.08 (Zerbino & Birney, 2008). The draft scaffolds were ordered and oriented by progressive Mauve genome alignment software with default settings (http://darlinglab.org/mauve/mauve.html). Gene prediction were performed using Glimmer 3.0 (Delcher, Bratke, Powers, & Salzberg, 2007), and *tRNA* and *rRNA* genes were predicted by *tRNA*scan‐SE (v1.21) (Lowe & Eddy, 1997) and RNAmmer (v1.2) (Lagesen et al., 2007), respectively. Predicted genes were annotated by GO database (Ashburner et al., 2000), KEGG pathway database (Kanehisa & Goto, 2000), COG functional classification system (Tatusov et al., 2003), and BLAST (http://blast.ncbi.nlm.nih.gov/Blast.cgi).

### 2.7 Statistical analysis

Data are presented as mean ± standard deviation (SD) of three independent biological replicates. Statistical comparisons were made by one‐way analysis of variance followed by Tukey's comparison test (XLstat software). A *p*‐value <.05 was considered to be significant.

## 3 RESULTS

### 3.1 Formation of VBNC state

**Figure 1:**
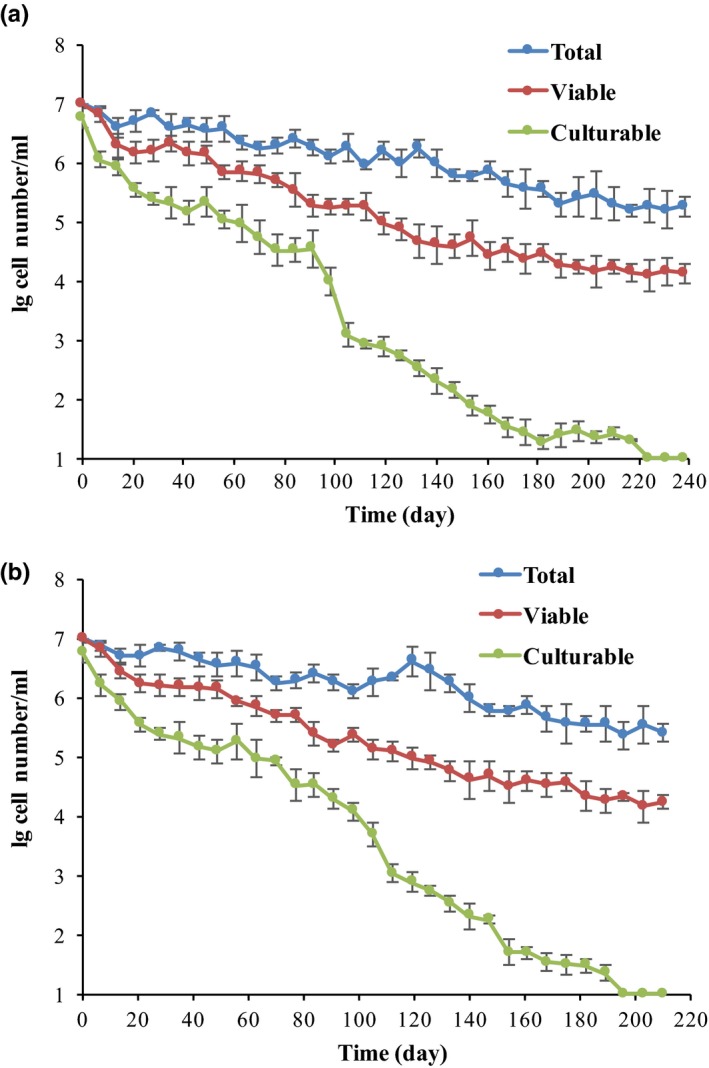
Entry of *Lactobacillus casei* BM‐LC14617 into the VBNC state upon beer subculture (A) or low‐temperature storage (0°C) (B), respectively

Cellular viability and culturability of *L*. *casei* strain were evaluated once a week, and the formation of VBNC state by *L*. *casei* was obtained and verified under both processes. According to the low‐temperature storage, none of *L*. *casei* cells was culturable after 196 ± 3.3 days, while viable cells revealed a low decrease with an approximate of 10^5^ cells/ml (Figure 1A). As for continuous passage in beer, 32 ± 1.6 subcultures were required for the formation of VBNC state of *L*. *casei* strains (Figure 1B).

### 3.2 Resuscitation of VBNC cells

When the VBNC cells induced by either adaptation protocol were subjected to addition of tween‐20, tween‐80, vitamin C, or vitamin B2, culturable cells were not obtained (data not shown). However, VBNC *L*. *casei* cells regained culturability on media containing catalase. Importantly, heat denaturation of catalase deferred the resuscitation process. The results observed here demonstrate that the addition of catalase is an effective method for the resuscitation of VBNC *L*. *casei* cells.

### 3.3 Beer‐spoilage ability

Exponentially growing, VBNC, and resuscitated cells were capable of maintaining in beer within approximately 10 days and further causing turbidity in beer (data not shown). It suggests the maintenance of beer‐spoilage capability of L. casei strain BM‐LC14617 under both VBNC and resuscitated states. After 30 days of incubation, increased levels of lactic acid and acetic acid were detected, which may eventually lead to beer acidization. Also, no significant difference was identified among the lactic acid, acetic acid, and diacetyl concentrations produced by exponentially growing, VBNC, and resuscitated cells, respectively. In addition, high level of diacetyl was also found under the VBNC states of *L*. *casei* strain, suggesting its attribution to imparting “buttery” off flavors in beer (Table 1). Detection of lactic acid, acetic acid, and diacetyl indicates that the VBNC and resuscitated *L*. *casei* cells remained viable and maintained similar beer‐spoilage capability as exponentially growing cells.

**Table 1:** Result of beer spoilage ability determination test

Strain	State	Turbidity	Diacetyl (mg/L)	Lactic acid (mg/L)	Acetic acid (mg/L)
*L*. *casei* BM‐LC14617	Exponentially growing	+	0.127	263.98	197.11
	VBNC	+	0.119	160.01	125.36
	Resuscitated	+	0.124	249.13	200.35

+: Positive.

### 3.4 Genomic feature

**Figure 2:**
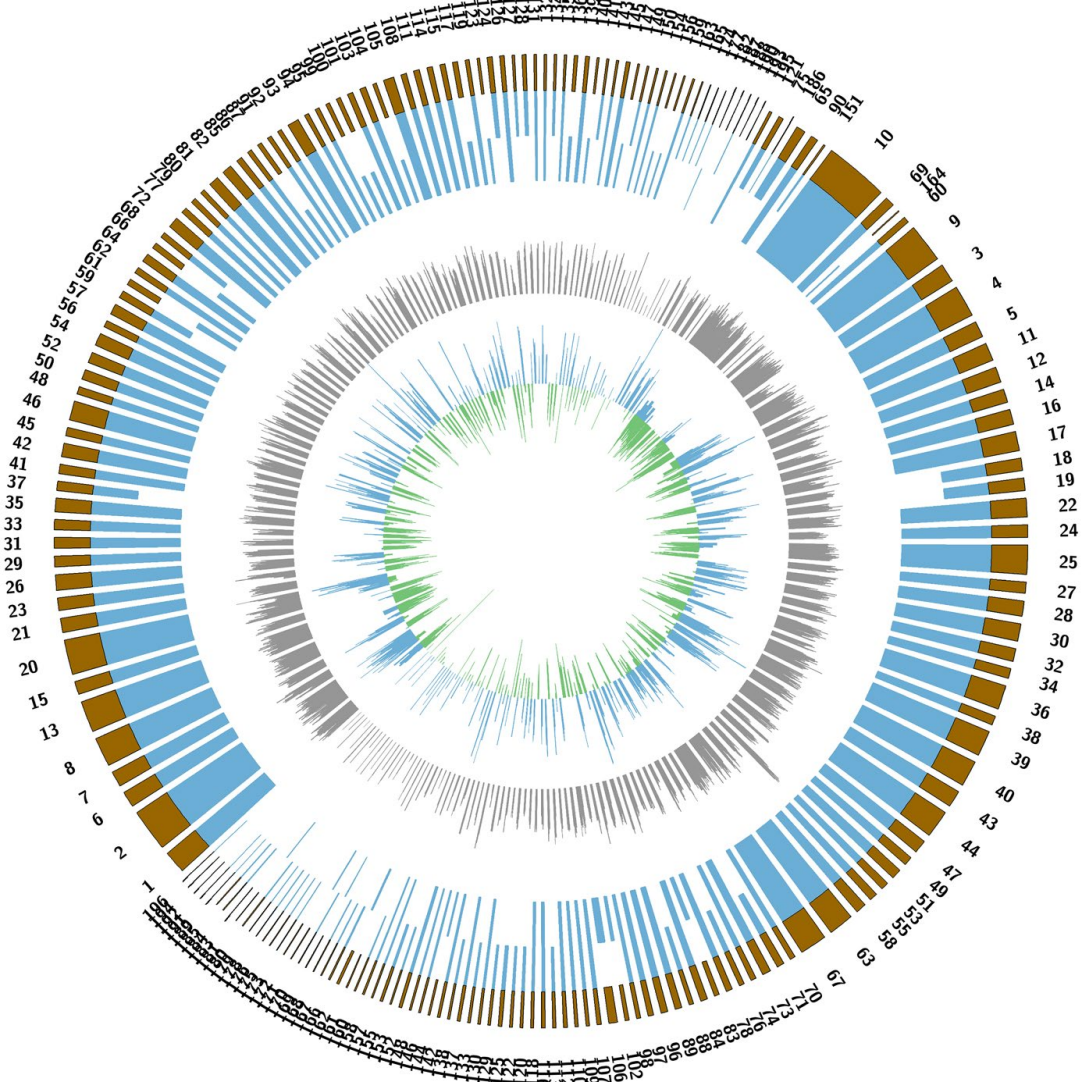
The genome circle of *L*. *casei* strain BM‐LA14617. The circle from outermost to innermost illustrates scaffold sequences, genes in plus strand, genes in minus strand, GC content, low GC content sequences, and high GC content sequences, respectively

**Figure 3:**
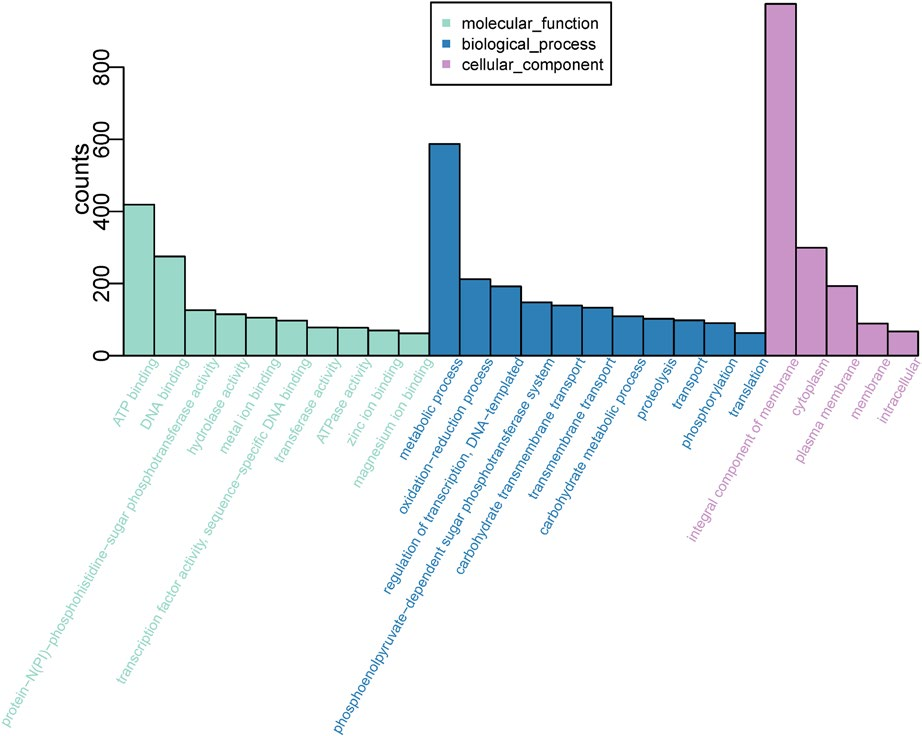
GO terms enrichment of genes in *L*. *casei* strain BM‐LC14617

**Figure 4:**
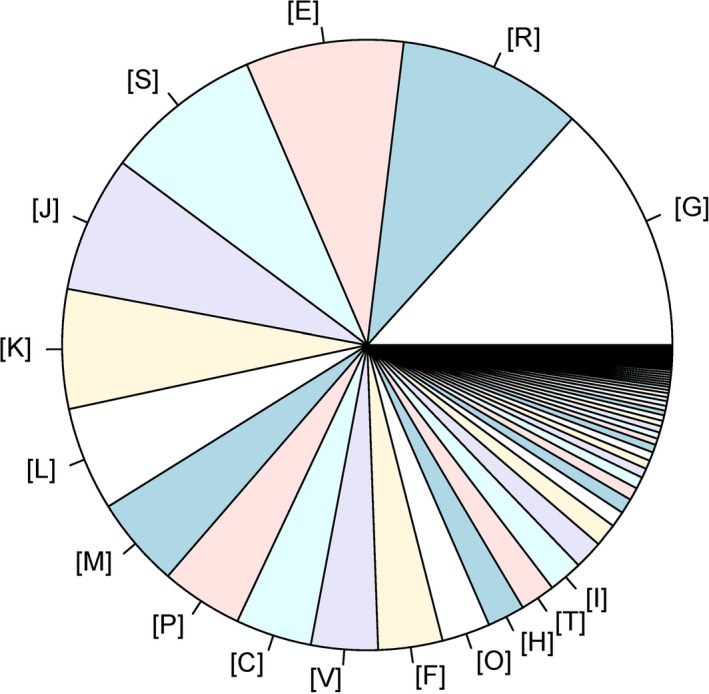
COG annotation of genes in *L*. *casei* strain BM‐LC14617. The numbers represent the number of genes in each COG category. [L]: DNA mismatch repair enzyme (predicted ATPase); [R]: General function prediction only; [E]: Amino acid transporters; [G]: Carbohydrate transport and metabolism; [K]: Transcriptional repressor of class III stress genes; [O]: Posttranslational modification, protein turnover, chaperones; [J]: Ribosomal protein L23; [C]: Acetate kinase; [T]: Signal transduction mechanisms; [P]: Inorganic ion transport and metabolism; [I]: Lipid transport and metabolism; [S]: Predicted membrane protein; [H]: Coenzyme transport and metabolism; [M]: Predicted outer membrane protein; [F]: Nucleotide transport and metabolism; [V]: Defense mechanisms

**Figure 5:**
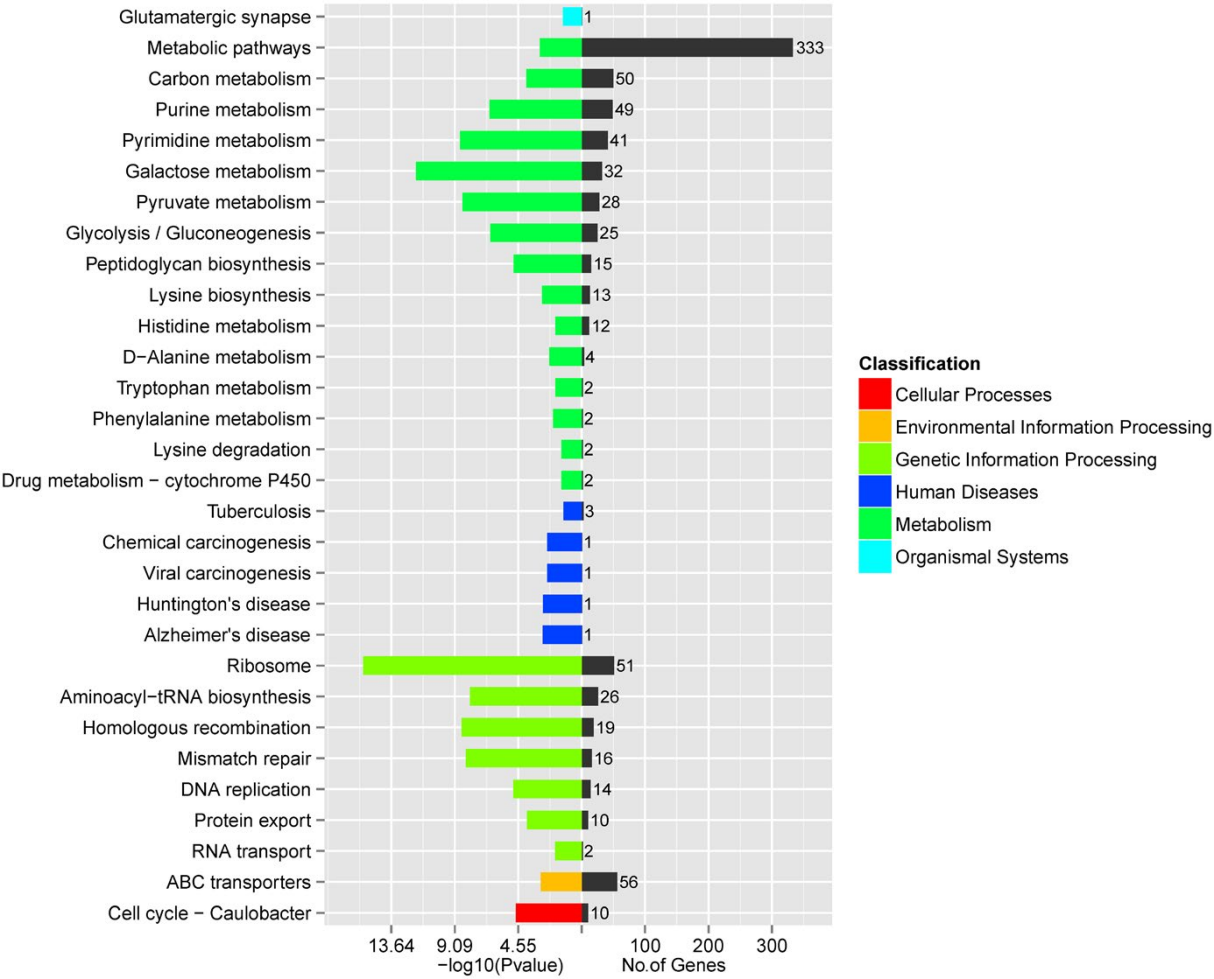
KEGG pathways enrichment results of genes in *L*. *casei* strain BM‐LC14617

The whole genomic sequence of beer‐spoilage *L*. *casei* strain BM‐LC14617 was obtained and further analyzed (Figure 2). The total length of the genome was 3,045,511 bp with a coverage of 99% and an average G + C content of 46.12%. The final assembly yielded 196 scaffolds, with the largest scaffold and N50 scaffold size found to be 128,069 bp and 26,583 bp in length, respectively. A total of 3,964 predicted genes with GO functional (Figure 3, Table S1), COG functional (Figure. 4, Table S2), and KEGG pathway (Figure 5, Table S3) annotations were identified, including one each for *23S rRNA* and *16S rRNA*, and 22 *tRNA* genes (24 RNA genes).

## 4 DISCUSSION

In this study, similar beer‐spoilage capability was found among exponentially growing, VBNC, and resuscitated *L*. *casei* cells, which was somewhat in accordance with previous studies (Buck & Oliver, 2010; Du et al., 2007; Imazaki & Nakaho, 2009; Jolivet‐Gougeon, Sauvager, Bonnaure‐Mallet, Colwell, & Cormier, 2006; Ordax, Biosca, Wimalajeewa, López, & Marco‐Noales, 2009; Patrone et al., 2013; Rahman, Suzuki, & Kawai, 2001). As beer brewing industry and *Lactobacillus* were concerned, *L. lindneri* and *L. paracollinoides* have been reported to be induced into the VBNC state by 30 subcultures in degassed beer and the VBNC cells showed beer‐spoilage capability (Suzuki et al., 2006). Also, formation of VBNC state for *L*. *acetotolerans* was found after 17 subcultures in beer or incubating in low‐temperature condition (Deng et al., 2015). In this study, The VBNC state *L*. *casei* strain BM‐LC14617 was obtained by continuous beer passages or low‐temperature storage, and the beer‐spoilage capability under VBNC state was verified. In addition, compared with *L. acetotolerans*, the shorter growth cycle (*L. acetotolerans* was hard to culture, requiring 14 days cultivation in MRS medium) (Deng et al., 2015), additional production of diacetyl, and higher frequency in beer‐spoilage cases for *L*. *casei* posed a significant concern for beer brewery industry. Nevertheless, with high identification rate in beer industry and capability of VBNC state formation under low‐temperature (common food storage condition), *L*. *casei* may be responsible for a large variety of beer‐spoilage cases.

As a beer‐spoilage bacterium, *L*. *casei* BM‐LC14617 was initially found to show relatively slow growth in MRS agar when isolated from beer sample, but after subculturing, this *L*. *casei* strain obtained culturability (data not shown). The addition of catalase is an effective method for the resuscitation of VBNC *L*. *casei* cells, and catalase addition has been known to improve flavor stability of beer in the brewing process (Festersen, Elvig, & Bisgaard‐Frantzen, 2007). Brewers can improve the microbiological quality control with significantly shorter incubation time by catalase supplementation. Low temperature (Deng et al., 2014; Griffitt, Noriea, Johnson, & Grimes, 2011; Imazaki & Nakaho, 2009; Kong et al., 2014; Zeng et al., 2013) or cold treatment combined with oligotrophic conditions (Chen et al., 2011; Dinu & Bach, 2011; Magajna & Schraft, 2015; Patrone et al., 2013; Sun et al., 2008; Zhong, Chen, Zhang, & Jiang, 2009) were frequently used to induce the VBNC state of various bacteria in previous studies. However, up to date, only three species of *Lactobacillus* strains have been verified for formation of VBNC state, including *L. lindneri*,* L. paracollinoides* (Suzuki et al., 2006), and *L. acetotolerans*, as induced to enter into the VBNC state by beer subculture treatment or low‐temperature storage (Deng et al., 2015). As a consequence, this study represented the first evidence on induction and resuscitation of the VBNC state by the species of *L*. *casei* strain.

As part of an initial strategy to investigate the phenotypic behavior of *L*. *casei*, the genomic features of *L*. *casei* strain BM‐LC14617 were investigated in this study via de novo genome sequencing. A variety of genes associated with VBNC state and beer‐spoilage were identified in the genome of *L*. *casei* BM‐LC14617. The adaption of bacteria to changing environment in order to survive under stress is a prerequisite for entry into the VBNC state. Predicted genes which were associated with defense (*n* = 44) and stress (*n* = 16) response were identified. In addition, nine predicted genes were involved in oxidative stress response which was reported to be associated with cold stress in bacteria (Chattopadhyay et al., 2011). Based on KEGG pathway database, predicted genes annotated to ABC transporters (*n* = 112) are essential for metabolism of nutrients and toxic molecules (Davidson, Dassa, Orelle, & Chen, 2008), which played a critical role in the bacterial adaptation to changing environment, survival under stress, as well as formation of VBNC state. Predicted genes encoding detoxification mechanism (*n* = 2) and associated with defense and resistance (antibiotics resistance, metal resistance, etc.) (*n* = 85) indicated the harmful substances exportation capability of *L*. *casei* strain thus survived under stress condition (Piddock, 2006). Moreover, additional predicted genes governing the VBNC state encode predicted membrane protein (*n* = 167), amino acid transporters (*n* = 163), transcriptional regulators (*n* = 138), and signal transduction (*n* = 44). Genes acting as transcriptional regulators and signal transducers enable bacteria to adapt to changing environments by rapid modification of cellular physiology, cell cycle progression, and development (Skerker, Prasol, Perchuk, Biondi, & Laub, 2005). Seven transcriptional regulators belong to MarR family involving in degradation of toxic environmental compounds, virulence, export of harmful chemicals, and resistance to oxidative stress (Grove, 2013), and four belongs to LysR family acting as an ortholog of OxyR in *Escherichia coli*. As a regulatory transcriptional factor sensitive to oxidation, OryR was capable of activating the expression of antioxidant genes in response to hydrogen peroxide in *E. coli* (Zheng, Aslund, & Storz, 1998). In addition, amino acids have been reported to be important growth substrates in bacteria and regulation on biosynthesis and degradation, and thus are essential for maintaining the carbon‐nitrogen balance in the VBNC cells (Postnikova, Shao, Mock, Baker, & Nemchinov, 2015). As far as beer spoilage is concerned, presence of genes involved in hop resistance, alcohol‐tolerance, and oxidative stress response are considered to be associated with the bacterial growth in beer, with one example being the correlation between carriage of *horA*,* horB,* and *horC* genes and hop resistance (DiMichele & Lewis, 1993; Suzuki et al., 2008). In the draft genome of *L*. *casei* BM‐LC14617, a homolog of the *Lactobacillus brevis* plasmid *horA* gene (GenBank accession no. AB005752.1) was identified, potentially explaining its affinity for growth in this medium. A cluster of genes encoding for alcohol‐tolerant enzymes (Brown, Guss, & Karpinets, 2011; Eanes, Merritt, Flowers, Kumagai, & Zhu, 2009; Ram, 2000), such as alcohol dehydrogenase (*n* = 12), aryl‐alcohol dehydrogenase (*n* = 2), malate dehydrogenase (*n* = 1), aldehyde dehydrogenase (*n* = 4), and glycerol‐3‐phosphate dehydrogenase (*n* = 3) were harbored by this genome. As a major beer‐spoilage compound, diacetyl is produced by dehydrogenation of 2,3‐butanediol (Siegel & Eggersdorfer, 2005). Acquiring (R,R)‐butanediol dehydrogenase activity, gene_3949 which might contribute to the diacetyl production, was identified in the genome of *L*. *casei* BM‐LC14617.

To date, a total of eight complete genome sequences of *L*. *casei* strains have been published. Among the eight *L*. *casei* strains, BL23, BD‐II, W56, and LOCK919 (85% coverage and 99% identity) showed the highest similarity with BM‐LC14617, followed by LC2W (84% coverage and 99% identity), 12A (84% coverage and 99% identity), Zhang (83% coverage and 99% identity), and ATCC334 (82% coverage and 99% identity). However, none of these *L*. *casei* strains were isolated from beer. Thus, this is the first genome sequence of beer‐spoilage *L*. *casei*. The difference between the genome of BM‐LC14617 and other *L*. *casei* strains (15%) might cover the major beer‐spoilage associated genes. Thus, the *L*. *casei* BM‐LC14617 specific sequences were further annotated and analyzed. Alcohol dehydrogenase and stress response encoding genes which might increase its alcohol tolerance and help surviving in beer were found. However, some function unknown genes might also play a role in the beer spoilage or VBNC state formation of *L*. *casei* BM‐LC14617.

## 5 CONCLUSION

This study focuses on the induction and resuscitation of VBNC state, beer‐spoilage ability, as well as the genomic analysis for *L*. *casei*. As for phenotype, the VBNC state of *L*. *casei* strain BM‐LC14617 had been induced by both beer subculture treatment and low‐temperature storage, and recovered by addition of catalase. Also, both VBNC and resuscitated cells remained beer‐spoilage capability. According to the phenotype, the genomic characterization of the beer‐spoilage *L*. *casei* strain BM‐LC14617 was also performed for insightful elucidation of VBNC state for *L*. *casei*. Information derived from this draft genome sequence may be relevant to study beer‐spoilage mechanisms in other lactic acid bacteria in an effort to reduce spoilage and improve food‐safety issues in the breweries. Further transcriptomic analysis and gene knockout studies will aid in additional insight into the genetic mechanisms that govern the VBNC state and beer‐spoilage capacity.

## NUCLEOTIDE SEQUENCE ACCESSION NUMBERS

The raw data and scaffold sequences of *L*. *casei* strain BM‐LA14617 were submitted to GenBank under accession number SRX1433289 and LTDP00000000, respectively.

## CONFLICT OF INTEREST

The authors have no conflict of interests to declare to this paper.

## References

[mbo3506-bib-0001] Ashburner, M. , Ball, C. A. , Blake, J. A. , Botstein, D. , Butler, H. , Cherry, J. M. , … Sherlock, G. (2000). Gene Ontology: Tool for the unification of biology. Nature Genetics, 25, 25–29. 10802651 10.1038/75556 PMC3037419

[mbo3506-bib-0002] Berney, M. , Hammes, F. , Bosshard, F. , Weilenmann, H. U. , & Egli, T. (2007). Assessment and interpretation of bacterial viability by using the LIVE/DEAD BacLight Kit in combination with flow cytometry. Applied and Environment Microbiology, 73, 3283–3290. 10.1128/AEM.02750-06 PMC1907116 17384309

[mbo3506-bib-0003] Brown, S. D. , Guss, A. M. , & Karpinets, T. V. (2011). Mutant alcohol dehydrogenase leads to improved ethanol tolerance in Clostridium thermocellum. Proceedings of the National Academy of Sciences of the United States of America, 108, 13752–13757. 21825121 10.1073/pnas.1102444108 PMC3158198

[mbo3506-bib-0004] Buck, A. , & Oliver, J. D. (2010). Survival of spinach‐associated *Helicobacter pylori* in the viable but nonculturable state. Food Control, 21, 1150–1154.

[mbo3506-bib-0005] Chattopadhyay, M. K. , Raghu, G. , Sharma, Y. V. R. K. , Biju, A. R. , Rajasekharan, M. V. , & Shivaji, S. (2011). Increase in oxidative stress at low temperature in an antarctic bacterium. Current Microbiology, 62, 544–546. 20730433 10.1007/s00284-010-9742-y

[mbo3506-bib-0006] Chen, H. , Fu, L. , Luo, L. , Lu, J. , White, W. L. , & Hu, Z. (2011). Induction and resuscitation of the viable but nonculturable state in a cyanobacteria‐lysing bacterium isolated from cyanobacterial bloom. Microbial Ecology, 63, 64–73. 21850447 10.1007/s00248-011-9928-2

[mbo3506-bib-0007] Davidson, A. L. , Dassa, E. , Orelle, C. , & Chen, J. (2008). Structure, function, and evolution of bacterial ATP‐binding cassette systems. Microbiology and Molecular Biology Reviews, 72, 317–364. 18535149 10.1128/MMBR.00031-07 PMC2415747

[mbo3506-bib-0008] Delcher, A. L. , Bratke, K. A. , Powers, E. C. , & Salzberg, S. L. (2007). Identifying bacterial genes and endosymbiont DNA with Glimmer. Bioinformatics, 23, 673–679. 17237039 10.1093/bioinformatics/btm009 PMC2387122

[mbo3506-bib-0009] Deng, Y. , Liu, J. , Li, L. , Fang, H. , Tu, J. , Li, B. , … Xu, Z. (2015). Reduction and restoration of culturability of beer‐stressed and low‐temperature‐stressed *Lactobacillus acetotolerans* strain 2011‐8. International Journal of Food Microbiology, 206, 96–101. 26001377 10.1016/j.ijfoodmicro.2015.04.046

[mbo3506-bib-0010] Deng, Y. , Liu, J. , Li, H. , Li, L. , Tu, J. , Fang, H. , … Qian, F. (2014). An improved plate culture procedure for the rapid detection of beer‐spoilage lactic acid bacteria. Journal of the Institute of Brewing, 120, 127–132.

[mbo3506-bib-0011] DiMichele, L. J. , & Lewis, M. J. (1993). Rapid species‐specific detection of lactic acid bacteria from beer using polymerase chain reaction. Journal of the American Society of Brewing Chemists, 51, 63–66.

[mbo3506-bib-0012] Dinu, L. D. , & Bach, S. (2011). Induction of viable but nonculturable *Escherichia coli* O157:H7 in the Phyllosphere of lettuce: A food safety risk factor. Applied and Environment Microbiology, 77, 8295–8302. 10.1128/AEM.05020-11 PMC3233046 21965401

[mbo3506-bib-0013] Du, M. , Chen, J. , Zhang, X. , Li, A. , Li, Y. , & Wang, Y. (2007). Retention of virulence in a viable but nonculturable *Edwardsiella tarda* isolate. Applied and Environment Microbiology, 73, 1349–1354. 10.1128/AEM.02243-06 PMC1828651 17189433

[mbo3506-bib-0014] Eanes, W. F. , Merritt, T. J. , Flowers, J. M. , Kumagai, S. , & Zhu, C. T. (2009). Direct evidence that genetic variation in glycerol‐3‐phosphate and malate dehydrogenase genes (Gpdh and Mdh1) affects adult ethanol tolerance in Drosophila melanogaster. Genetics, 181, 607–614. 19033156 10.1534/genetics.108.089383 PMC2644950

[mbo3506-bib-0015] Festersen, M. R. , Elvig, N. , & Bisgaard‐Frantzen, H . (2007) Beer‐brewing method. WIPO Patent Application WO/2007/101846.

[mbo3506-bib-0016] Griffitt, K. J. , Noriea, N. F. , Johnson, C. N. , & Grimes, D. J. (2011). Enumeration of *Vibrio parahaemolyticus* in the viable but nonculturable state using direct plate counts and recognition of individual gene fluorescence in situ hybridization. Journal of Microbiol Methods, 85, 114–118. 10.1016/j.mimet.2011.02.006 21329738

[mbo3506-bib-0017] Grove, A. (2013). *MarR* family transcription factors. Current Biology, 23, R142–R143. 23428319 10.1016/j.cub.2013.01.013

[mbo3506-bib-0018] Hobbie, J. E. , Daley, R. J. , & Jasper, S. (1977). Use of nucleopore filters for counting bacteria by fluorescence microscopy. Applied and Environment Microbiology, 33, 1225–1228. 10.1128/aem.33.5.1225-1228.1977 PMC170856 327932

[mbo3506-bib-0019] Imazaki, I. , & Nakaho, K. (2009). Temperature‐upshift‐mediated revival from the sodium‐pyruvate‐recoverable viable but nonculturable state induced by low temperature in *Ralstonia solanacearum*: Linear regression analysis. Journal of General Plant Pathology, 75, 213–226.

[mbo3506-bib-0020] Jolivet‐Gougeon, A. , Sauvager, F. , Bonnaure‐Mallet, M. , Colwell, R. R. , & Cormier, M. (2006). Virulence of viable but nonculturable *S. Typhimurium* LT2 after peracetic acid treatment. International Journal of Food Microbiology, 112, 147–152. 16876276 10.1016/j.ijfoodmicro.2006.06.019

[mbo3506-bib-0021] Kanehisa, M. , & Goto, S. (2000). KEGG: Kyoto encyclopedia of genes and genomes. Nucleic Acids Research, 28, 27–30. 10592173 10.1093/nar/28.1.27 PMC102409

[mbo3506-bib-0022] Kong, H. G. , Bae, J. Y. , Lee, H. J. , Joo, H. J. , Jung, E. J. , Chung, E. , & Lee, S. W. (2014). Induction of the viable but nonculturable state of *Ralstonia solanacearum* by low temperature in the soil microcosm and its resuscitation by catalase. PLoS ONE, 9, e109792. 25296177 10.1371/journal.pone.0109792 PMC4190316

[mbo3506-bib-0023] Lagesen, K. , Hallin, P. , Rodland, E. A. , Staerfeldt, H. H. , Rognes, T. , & Ussery, D. W. (2007). RNAmmer: Consistent and rapid annotation of ribosomal RNA genes. Nucleic Acids Research, 35, 3100–3108. 17452365 10.1093/nar/gkm160 PMC1888812

[mbo3506-bib-0024] Lowe, T. M. , & Eddy, S. R. (1997). tRNAscan‐SE: A program for improved detection of transfer RNA genes in genomic sequence. Nucleic Acids Research, 25, 955–964. 9023104 10.1093/nar/25.5.955 PMC146525

[mbo3506-bib-0025] Magajna, B. A. , & Schraft, H. (2015). *Campylobacter jejuni* biofilm cells become viable but non‐culturable (VBNC) in low nutrient conditions at 4°C more quickly than their planktonic counterparts. Food Control, 50, 45–50.

[mbo3506-bib-0026] Oliver, J. D. (2005). The viable but nonculturable state in bacteria. Journal of Microbiology, 43, 93–100. 15765062

[mbo3506-bib-0027] Ordax, M. , Biosca, E. G. , Wimalajeewa, S. C. , López, M. M. , & Marco‐Noales, E. (2009). Survival of *Erwinia amylovorain* mature apple fruit calyces through the viable but nonculturable (VBNC) state. Journal of Applied Microbiology, 107, 106–116. 19298508 10.1111/j.1365-2672.2009.04187.x

[mbo3506-bib-0028] Patrone, V. , Campana, R. , Vallorani, L. , Dominici, S. , Federici, S. , Casadei, L. , … Baffone, W. (2013). *CadF* expression in *Campylobacter jejuni* strains incubated under low‐temperature water microcosm conditions which induce the viable but non‐culturable (VBNC) state. Antonie van Leeuwenhoek, 103, 979–988. 23314927 10.1007/s10482-013-9877-5

[mbo3506-bib-0029] Piddock, L. J. (2006). Multidrug‐resistance efflux pumps‐not just for resistance. Nature Reviews Microbiology, 4, 629–636. 16845433 10.1038/nrmicro1464

[mbo3506-bib-0030] Postnikova, O. A. , Shao, J. , Mock, N. M. , Baker, C. J. , & Nemchinov, L. G. (2015). Gene expression profiling in viable but nonculturable (VBNC) cells of *Pseudomonas syringae pv syringae* . Frontiers in Microbiology, 6, 1419. 26733964 10.3389/fmicb.2015.01419 PMC4683178

[mbo3506-bib-0031] Rahman, M. H. , Suzuki, S. , & Kawai, K. (2001). Formation of viable but non‐culturable state (VBNC) of *Aeromonas hydrophila* and its virulence in goldfish, Carassius auratus. Microbiological Research, 156, 103–106. 11372646 10.1078/0944-5013-00084

[mbo3506-bib-0032] Ram, S. (2000). Role of alcohol dehydrogenase, malate dehydrogenase and malic enzyme in flooding tolerance in Brachiaria species. Journal of Plant Biochemistry and Biotechnology, 9, 45–47.

[mbo3506-bib-0033] Sakamoto, K. , & Konings, W. N. (2003). Beer spoilage bacteria and hop resistance. International Journal of Food Microbiology, 89, 105–124. 14623377 10.1016/s0168-1605(03)00153-3

[mbo3506-bib-0034] Siegel, H. , & Eggersdorfer, M. (2005). Ullmann's Encyclopedia of Industrial Chemistry. Weinheim: Wiley‐VCH.

[mbo3506-bib-0035] Skerker, J. M. , Prasol, M. S. , Perchuk, B. S. , Biondi, E. G. , & Laub, M. T. (2005). Two‐component signal transduction pathways regulating growth and cell cycle progression in a bacterium: A system‐level analysis. PLoS Biology, 3, e334. 16176121 10.1371/journal.pbio.0030334 PMC1233412

[mbo3506-bib-0036] Sun, F. , Chen, J. , Zhong, L. , Zhang, X. H. , Wang, R. , Guo, Q. , & Dong, Y. (2008). Characterization and virulence retention of viable but nonculturable *Vibrio harveyi* . FEMS Microbiology Ecology, 64, 37–44. 18248440 10.1111/j.1574-6941.2008.00442.x

[mbo3506-bib-0037] Suzuki, K. , Asano, S. , Iijima, K. , & Kitamoto, K. (2008). Sake and beer spoilage lactic acid bacteria‐a review. Journal of the Institute of Brewing, 114, 209–223.

[mbo3506-bib-0038] Suzuki, K. , Iijima, K. , Asano, S. , Kuriyama, H. , & Kitagawa, Y. (2006). Induction of viable but nonculturable state in beer spoilage lactic acid bacteria. Journal of the Institute of Brewing, 112, 295–301.

[mbo3506-bib-0039] Tatusov, R. L. , Fedorova, N. D. , Jackson, J. D. , Jacobs, A. R. , Kiryutin, B. , Koonin, E. V. , … Natale, D. A. (2003). The COG database: An updated version includes eukaryotes. BMC Bioinformatics, 4, 41. 12969510 10.1186/1471-2105-4-41 PMC222959

[mbo3506-bib-0040] Zeng, B. , Zhao, G. , Cao, X. , Yang, Z. , Wang, C. , & Hou, L. (2013). Formation and resuscitation of viable but nonculturable *Salmonella typhi* . BioMed Research International, 2013, 907170. 23509799 10.1155/2013/907170 PMC3591152

[mbo3506-bib-0041] Zerbino, D. R. , & Birney, E. (2008). Velvet: Algorithms for de novo short read assembly using de Bruijn graphs. Genome Research, 18, 821–829. 18349386 10.1101/gr.074492.107 PMC2336801

[mbo3506-bib-0042] Zheng, M. , Aslund, F. , & Storz, G. (1998). Activation of the OxyR transcription factor by reversible disulfide bond formation. Science, 279, 1718–1721. 9497290 10.1126/science.279.5357.1718

[mbo3506-bib-0043] Zhong, L. , Chen, J. , Zhang, X. , & Jiang, Y. (2009). Entry of *Vibrio cincinnatiensisinto* viable but nonculturable state and its resuscitation. Letters in Applied Microbiology, 48, 247–252. 19196443 10.1111/j.1472-765X.2008.02522.x

